# Understanding the ablation rate of Holmium:YAG and thulium fiber lasers. Perspectives from an in vitro study

**DOI:** 10.1007/s00240-022-01402-6

**Published:** 2023-01-17

**Authors:** Mario Basulto-Martínez, Silvia Proietti, Maria Pia Pavia, Yuyi Yeow, Brian H. Eisner, Guido Giusti

**Affiliations:** 1 Department of Urology, IRCCS San Raffaele Hospital, Milan, Italy; 2 European Training Center in Endourology, Milan, Italy; 3 Department of Urology, Hospital Regional de Alta Especialidad de la Península de Yucatán, Merida, Mexico; 4 Department of Urology, “Ospedale Riuniti” University Hospital, Marche Polytechnic University, Ancona, Italy; 5 Department of Urology, Massachusetts General Hospital, Boston, MA USA; 6 Istituto di Ricovero e Cura a Carattere Scientifico, Ospedale San Raffaele S.r.l., Via Olgettina 60, 20132 Milan, MI Italy

**Keywords:** Kidney stones, Urolithiasis, Lithotripsy, Holmium laser, Thulium fiber laser

## Abstract

This study sought to analyze the ablation rates of Holmium:YAG (Ho:YAG) and thulium fiber laser (TFL) under different settings combinations. Lasering was carried out by a computed spiral pattern through a stepper motor bearing a laser fiber. BegoStones were placed inside a water container and lasered therein and the ablation rate was calculated. Different combinations were tested for ~12 Watts (W) for Ho:YAG and TFL lasers. Further 25 W combination for TFL were conducted testing higher frequencies. One-hundred-one experiments were conducted. Under ~12 W, the highest ablation rate for Ho:YAG was obtained with 40 Hz/0.3 J, and Virtual Basket™ (VB) emission mode [114.35 (88.30 – 126.40) mg/min] whereas for TFL, using 40 Hz/0.3 J, and long pulse [143.40 (137.40 – 146) mg/min]. A matched-comparison for 12 Hz/1 J settings showed that TFL outperformed Ho:YAG using medium pulse [63.30 (55.30 – 81.30) vs 132.00 (115.70 – 228.60) mg/min, p=0.016] and long pulse [62.90 (51.60 – 78.90) vs 134.70 (110.60 – 149.30) mg/min, p=0.016]. In the 40 Hz/0.3 J settings, TFL outperformed Ho:YAG (p=0.034). Nonetheless, Ho:YAG with VB’s ablation rate increased [114.35 (88.30 – 126.40)], being comparable to that of TFL with medium pulse [127.5 (88.90 – 141.70)] (p=0.400). In TFL 25 W experiments, the ablation rate of 500 Hz / 0.05 J, was higher than those of 1000 Hz / 0.025 J (p=0.049). TFL ablation rate is higher than that of Ho:YAG. Moreover, the Virtual Basket™ emission mode, increased Ho:YAG ablation rates, resulting reaching similar to that of TFL in certain modalities.

**Supplementary Information:**

The online version contains supplementary material available at 10.1007/s00240-022-01402-6.

## Introduction

The laser technology has revolutionized kidney stones treatment in the last decades. Holmium: yttrium aluminum garnet (Ho:YAG) is the gold standard device for lithotripsy, and can be safely and effectively used within the urinary for all types of stones [1, 2]. Ho:YAG is a 2.1 µm wavelength solid-state pulsed laser, highly absorbed in water. The main ablative mechanism of Ho:YAG is by photothermal effect [3]; as the energy delivered is absorbed by the stones causing its breakage [4]. Different Ho:YAG devices commercialized displays diverse features, like total power output regulation and pulse width modulation, allowing diverse settings for better performance [1].

Furthermore, manufacturers commercialize laser generators with technology able to provide pulse delivery modes, improving energy transmission. The Moses™ technology (Lumenis^®^, Yokneam, Israel), is a modulated pulse mode in which energy is released into two peaks, the first creates the water cavity known as the Moses effect [5] so that the second reaches the target stone more effectively [6]. This technology has demonstrated better ablation and less retropulsion both in vitro [6] and in a clinical trial [7]. Another similar emission mode named Virtual Basket™ (VB) (Quanta System, Samarate, Italy) has been developed. This pulse-shape modulation delivers an initial pulse creating a first bubble, then delivering a second pulse when the first bubble reaches its maximum expansion. This double-pulse modulation aims to generate a more efficient communication channel with the target stone. Researchers have communicated improved ablation and lower retropulsion using the VB modality [1, 8–10].

Thulium fiber laser (TFL) operates at a 1.9 µm wavelength, nearly matching water’s absorption peak, becoming four times higher than that of Ho:YAG. Furthermore, TFL may reach higher pulse frequencies than Ho:YAG, beyond 2000 Hz, and has therefore gained attention as a strong competitor for lithotripsy due to its faster ablation and finer pulverization. [4, 11, 12]. Moreover, the VB’s bubble dynamics has been suggested as an enhancer of energy delivery, however, it has not been observed in vitro. In this study we aimed to compare the ablation rates using different settings and power outputs for TFL and Ho:YAG, including VB.

## Material and methods

### Sample stones

Stones were created simulating calcium oxalate monohydrate stones using a mixture of 100 mg of BegoStone and 20 mL of water [13, 14] and then cast into a mold to let dry for 24 h obtaining flat disks of 20 mm diameter, 4 mm thick, calculate volume of 1.25 cm^3^, and 3.9 ± 0.1 g weight. Each stone was weighted (Kern TAB, Ken, Germany) and then soaked in tap water for rehydration for 15 min before each experiment.

### Experimental setup

**Fig. 1:**
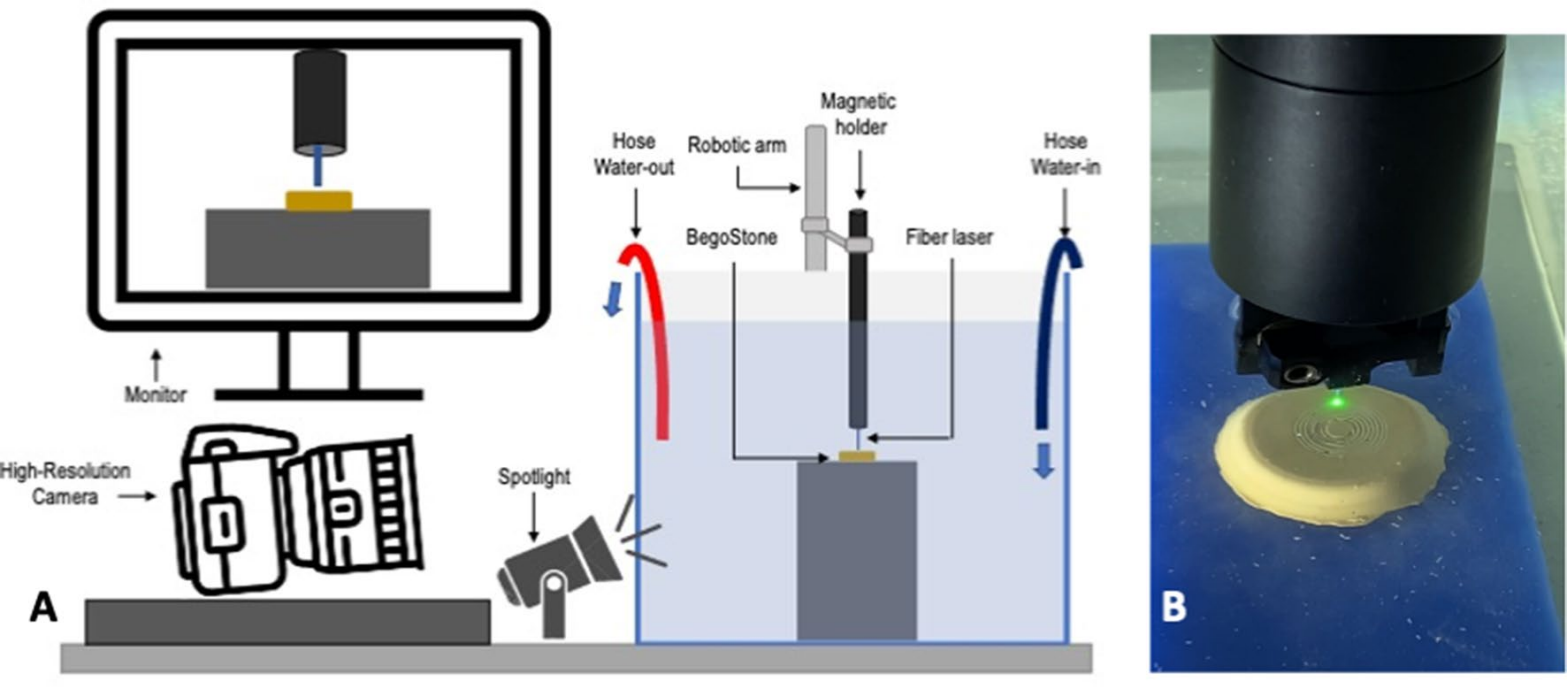
Main experimental setup. **A** Displays the schematic setup, in which a water container under continuous irrigation with an inflow hose at 1.2 mL/s with a pump (not shown), and an outflow hose, containing a flat BegoStone. The magnetic holder holding the fiber and controlled by a robotic arm and a stepper motor (not shown). All experiments were visually controlled with a high-resolution camera connected to a monitor; **B** shows an experiment close-up picture

**Fig. 2:**
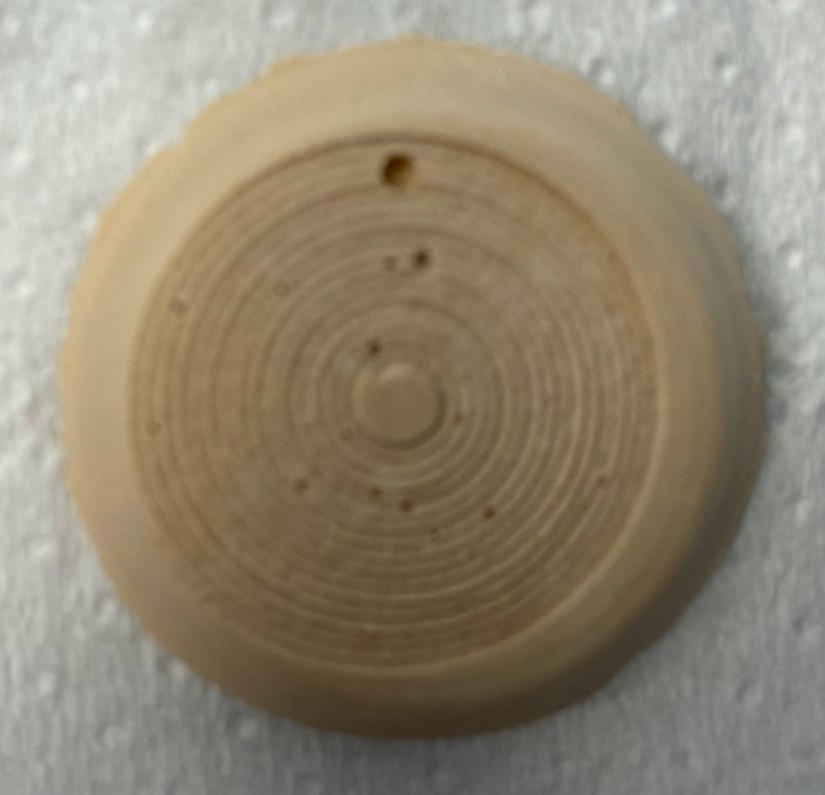
Ablated BegoStone with a spiral pattern footprint

Lasering was conducted using a stepper motor with a computed preset spiral pattern spinning at 5 mm/s. The laser fiber was held into the sliding arm using a magnetic holder and placed with the tip 0.5 mm away from the targeted stone. The stone was placed over a flat surface and immersed into a water container, with a continuous irrigation system set a *t* 1.2 mL/s. The laser was fired over the flat stone until the spiral was completed and was visually controlled with a high-resolution camera (AX200 FASTCAM Mini AX200, Photron, Japan). The experimental setup is displayed in Fig. 1. The ablation time (s) was recorded, and the ablation footprint of each stone was examined to rule out any unevenly fired stone (Fig. 2). Before every set of experiments, the laser fiber was peeled and cut following the manufacturer’s recommendation using a ceramic cutter and a fiber stripper (Quanta System, Samarate, Italy); and the laser power output was confirmed using a power meter (Nova II, Ophir, Israel). The ablated stones were let dry for 24 h and then weighted. The mass loss (mg) was recorded by subtracting it from baseline dry weight. The ablation rate was calculated dividing mass loss by exposure time (mg/s).

### Laser devices and settings

All experiments were conducted either with a Cyber Ho 150 Watts (W) (Ho:YAG, Quanta System, Samarate, Italy) or a Fiber Dust™ (TFL, Quanta System, Samarate, Italy) using a 272 µm core fiber (Quanta System, Samarate, Italy). Different settings combinations for 12–12.5 W were tested, as follows: Ho:YAG 12 Hz/1 J with short (SP), medium (MP), and long pulse (LP), as well as VB; 40 Hz/0.3 J with SP, MP, LP, and VB; 25 Hz/0.5 J SP and VB; and 15 Hz/0.8 J SP and VB. TFL 12 Hz/1 J SP, MP, and LP; 40 Hz/0.3 J SP and MP; 120 Hz/0.1 J SP, MP, and LP; and 500 Hz/0.025 J MP. Similarly, we tested four combinations of 25 W for the TFL as follows: 500 Hz/0.05 J, SP and LP; and 1000 Hz/0.025 J, MP and LP. For TFL with SP settings, the peak power was ~ 500 W for all combinations, to explore higher frequencies unreachable with a ~ 12 W output power.

### Statistical analysis

Median and ranges of the ablation rates were compared using either *U*-Mann–Whitney or Kruskal–Wallis’s test as appropriate, with Bonferroni’s correction. The analyses were conducted with SPSS^®^ v.25 (IBM^®^, USA). *P*-values < 0.05 were considered as statistically significant. Due to the nature of the experiment, the Institutional Review Board approval was waived.

## Results

**Fig. 3:**
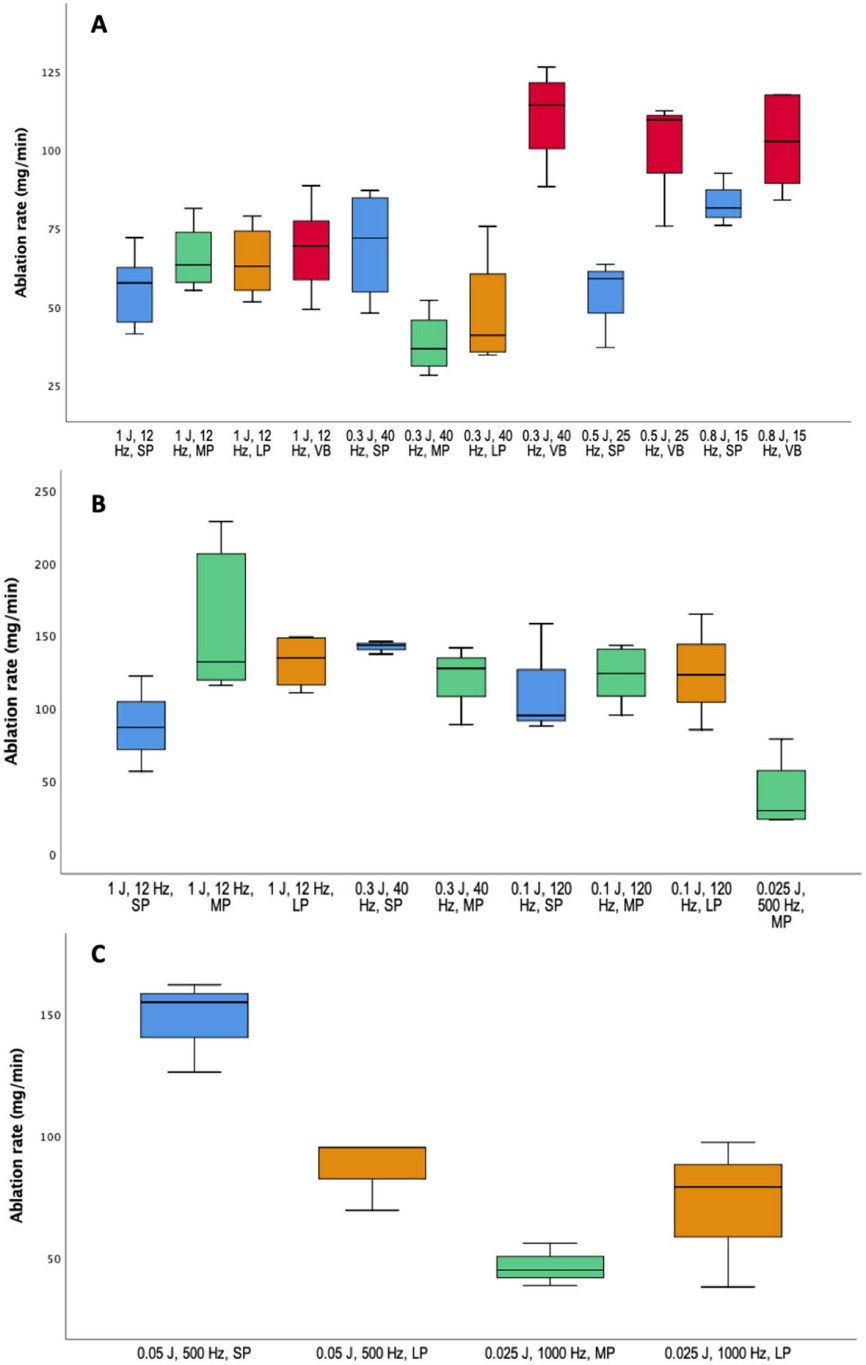
Ablation rates for **A** Ho:YAG 12–12.5 W; **B** TFL 12–12.5 W; and TFL 25 W. Box colors are as following: blue, short pulse; green, medium pulse; orange, long pulse; and red, virtual basket

A total of 101 experiments were conducted. Each combination was repeated four times on average. Each setting tested and their ablation rates are enlisted in Table 1. For the experiments with a total output power of 12–12.5 W, twenty-one setting combinations were tested, twelve for Ho:YAG and nine for TFL, and are presented in Fig. 3A, B, respectively. The highest ablation rate for Ho:YAG was 114.35 (88.30–126.40) mg/min, achieved using 40 Hz/0.3 J, with VB; whereas for TFL was 143.40 (137.40–146) mg/min, with 40 Hz/0.3 J, with LP; both ablation rates were comparable with a borderline statistical significance (*p* = 0.057). None of the bubble’s length was shorter than 0.5 mm, as their size ranged from 1.15 to 4.11 mm.

**Table 1:** Energy settings and ablation rates of the experiments conducted with Ho:YAG and TFL lasers

Laser	Pulse frequency (Hz)	Pulse energy (J)	Total power output (W)	Pulse mode	*n*	Mass loss (g) Median (25–75 percentile)	Exposure time (s) Median (25–75 percentile)	Ablation rate, median (range)
Ho:YAG	12	1	12	SP	6	84.8 (77.5–92)	92 (78.7–111.7)	57.65 (41.30–72.10)
Ho:YAG	12	1	12	MP	4	110.5 (90.3–140.7)	107.5 (90–113)	63.30 (55.30–81.30)
Ho:YAG	12	1	12	LP	5	119.8 (106.8–139.7)	110 (105.5–127)	62.90 (51.60–78.90)
Ho:YAG	12	1	12	VB	9	124.4 (97.9–150.1)	111 (103.5–114)	69.40 (49.20–88.60)
TFL	12	1	12	SP	3	157.6 (80.2–157.6)	108 (85–108)	86.80 (56.60 –122.20)
TFL	12	1	12	MP	5	275 (234.8–432.6)	125 (111–128)	132.00 (115.70–228.60)
TFL	12	1	12	LP	4	215.9 (212.2–113.2)	106.5 (92.2–113.2)	134.70 (110.60–149.30)
Ho:YAG	40	0.3	12	SP	4	145.4 (95.5–181.5)	120.5 (111.2–126.7)	71.95 (48–87.10)
Ho:YAG	40	0.3	12	MP	4	71.5 (53–96.7)	115.5 (102.5–124)	36.70 (28.20–52.10)
Ho:YAG	40	0.3	12	LP	4	70.7 (61.5–127.6)	107 (98.5–114.7)	40.95 (34.70–75.70)
Ho:YAG	40	0.3	12	VB	4	227.2 (194.2–243.5)	122.5 (111.7–127.2)	114.35 (88.30–126.40)
TFL	40	0.3	12	SP	3	239 (197–239)	100 (86–100)	143.40 (137.40–146)
TFL	40	0.3	12	MP	3	248 (163–248)	110 (105 –110)	127.5 (88.90–141.70)
Ho:YAG	25	0.5	12.5	SP	3	118 (60–118)	120 (110–120)	59 (37.10–63.50)
Ho:YAG	25	0.5	12.5	VB	3	195 (144–195)	110 (104–110)	109.60 (75.80–112.50)
Ho:YAG	15	0.8	12	SP	4	153.5 (130.2–170.7)	114.5 (96–118.7)	81.45 (76.0–92.60)
Ho:YAG	15	0.8	12	VB	6	169 (135–191.5)	98.5 (90–100)	102.70 (84–117.6)
TFL	120	0.1	12	SP	3	180.6 (139–180.6)	101 (95–101)	95.10 (87.80–158.30)
TFL	120	0.1	12	MP	5	213 (190.7–242.6)	104 (102–112)	124.10 (95.40–143.40)
TFL	120	0.1	12	LP	3	236 (139.4–236)	115 (98–115)	123.10 (85.30–164.90)
TFL	500	0.025	12.5	MP	4	52.5 (41.1–111.3)	104 (98.2–108.2)	29.60 (23.40–78.90)
TFL	500	0.05	25	SP	3	273 (242–273)	115 (101–115)	155 (126.30–162.2)
TFL	500	0.05	25	LP	3	148 (130–148)	110 (93–110)	95.50 (69.60–95.50)
TFL	1000	0.025	25	MP	3	67.4 (55–67.4)	85 (72–85)	45.10 (38.80–56.20)
TFL	1000	0.025	25	LP	3	165 (51–165)	120 (80–120)	79.20 (38.20–97.50)

*g* grams, *Ho:YAG* holium:YAG, *LP* long pulse, *MP* medium pulse, *s* seconds, *SP* short pulse, *TFL* thulium fiber laser, *VB* virtual basket™

The ablation rates obtained with 12 Hz/1 J and different pulse widths were similar for Ho:YAG (*p* = 0.375) as well as for TFL using the same combinations (*p* = 0.196). However, a matching comparison showed that ablation rates were only similar between Ho:YAG and TFL in SP [57.65 (41.30–72.10) vs 86.80 (56.60–122.20), *p* = 0.167], but significantly higher for TFL in MP [63.30 (55.30–81.30) vs 132.00 (115.70–228.60), *p* = 0.016] and LP [62.90 (51.60–78.90) vs 134.70 (110.60–149.30), *p* = 0.016]. In the 40 Hz/0.3 J settings, TFL outperformed Ho:YAG (*p* = 0.034). Nonetheless, the Ho:YAG with VB’s ablation rate increased substantially [114.35 (88.30–126.40)], resulting comparable to that of TFL with MP [127.5 (88.90–141.70)] (*p* = 0.400). The ratios between standard pulse modulation (SP) and VB, with respect to the different settings combinations for Ho:YAG, are summarized in Table 2. Notably, the ablation rates with VB were significantly higher for 40 Hz/0.3 J and 25 Hz/0.5 J (*p* = 0.021 and *p* = 0.05, respectively). In the TFL 25 W experiments, the ablation rate of 500 Hz/0.05 J, was higher than those of 1000 Hz/0.025 J (*p* = 0.049) (Fig. 3C).

**Table 2:** Comparison between ablation rates with VB and SP with Ho:YAG laser

Settings	Ablation rate median (range) mg/min		Ratio	Improvement (%)	*p*
	SP	VB			
12 Hz/1 J	57.6 (41.3–72.1)	69.4 (49.2–88.6)	1:1.20	20.5	0.126
15 Hz/0.8 J	81.4 (76.0–92.6)	102.7 (84.0–117.6)	1:1.26	26	0.055
25 Hz/0.5 J	59.0 (37.1–63.5)	109.6 (75.8–112.5)	1:1.85	85.7	0.049*
40 Hz/0.3 J	71.9 (48.0–85.9)	114.3 (88.3–126.4)	1:1.59	59	0.021*

*SP* short pulse, *VB* virtual basket™

*Statistically significant

## Discussion

In this in vitro study, we evaluated the ablation rates of Ho:YAG and TFL using different settings for a total power output of 12–12.5 W, and tested further experiments with 25 W for TFL. The ablation rates increased as the pulse width lengthened for Ho:YAG, as previously reported in similarly-conducted experiments [13], reaching its highest with the VB modality. Overall, TFL ablation rates were higher, at least doubling those of Ho:YAG (Table 1).

In the 12 Hz/1 J matching analysis, TFL outperformed Ho:YAG in all pulse widths, although for SP was not statistically significant (*p* = 0.167). Despite Ho:YAG with VB improved the ablation rate, it was not as high as those of TFL. Considering the reproducible and standardized methodology developed, along with the paired energy settings, it is strongly suggested that the TFL wavelength itself may achieve higher ablation rates. A possible explanation for this finding is the TFL wavelength’s higher water absorption coefficient compared to Ho:YAG wavelength, theoretically improving ablation by photothermal and thermomechanical effects [4, 15]. Similar results have been communicated. Panthier et al. [14], using 15 Hz/1 J (15 W) reported an ablation rate for Ho:YAG and TFL of 31.74 ± 4.60 and 66.96 ± 11.39 mm^3^/min, respectively; pulse width was not disclosed. Notably, TFL also doubled Ho:YAG ablation rate. The fact that the authors reported lower ablation rates than those in the current study, despite similar setup, fiber core diameter, higher power output (12 and 15 W), and contact mode (0.5 mm away in current work) might be explained by the ablation rate measurement, that was estimated as volume loss, but a good correlation was reported to the grams of mass loss (*r* = 0.72). Another possible factor is the spatial beam, which has been reported as multimodal or with ‘hot-spots’ in Ho:YAG whereas is undeviating in TFL, delivering energy more effectively [14, 16]. It has been postulated that the better ablation rate of TFL is partly achieved by reducing retropulsion, as the pressure emitted from fiber tip is lower and the bubbles created are smaller than those of Ho:YAG [17], however, this would not play a role in this experimental model where stones are fixed into a flat surface hindering backward displacement but it would in a real clinical scenario.

Similarly, in the 40 Hz/0.3 J setting, ablation rates increased with respect to 12 Hz/1 J on both lasers, suggesting that a more efficient ablation is reached with this modality. Again, TFL at least doubled Ho:YAG’s ablation rates. Noteworthy, the Ho:YAG with VB reached an ablation rate comparable to that of TFL. Hardy et al. [18], compared the ablation rate of Ho:YAG and TFL using human calcium oxalate monohydrate kidney stones. Researchers tested three modalities arranged as follows G1 [10 W: 50 Hz/0.2 J, (SP, TFL: 500 µs; Ho:YAG: 200 µs)], G2 [16 W: 50 Hz/0.2 J, (SP, TFL: 500 µs; Ho:YAG: 200 µs)], and G3 [32 W: 80 Hz/0.4 J, (LP, TFL: 1000 µs; and MP, Ho:YAG: 350 µs)]. The ablation rates obtained were higher for TFL in the G1 (18 ± 12 vs 48 ± 12 mg/min); G2 (36 ± 6 vs 60 ± 24 mg/min) and G3 (42 ± 12 vs 78 ± 54 mg/min). As compared to our results, ablation rates were lower and this is possibly explained by the fact that human stones are not as homogenous as BegoStones, and its surface is irregular, bringing constant changes in the stone-fiber distance dissipating more energy. In addition, a study [19] reported similar results, with TFL achieving ablation rates twofold or threefold over Ho:YAG, depending on stone hardness. A clinical trial [20] compared patients with ureteral stones randomized either for TFL or Ho:YAG, using 10 Hz/1 J settings, and a shorter lasering time was observed for TFL (8.4 ± 0.4 vs 15.9 ± 0.5 min, *p* < 0.05).

The ablation rates improved with VB emission mode as compared to SP (Table 2). This might be explained by the VB bubble elongation and more effective energy delivery. Moreover, LP had a higher ablation rate than SP using 1 J pulse energy, however, this was the opposite for lower pulse energy. These data suggest that the use of high frequency as in dusting, should be ideally performed with SP or VB to reach better ablation rates. The shorter bubbles in TFL result from the lower peak power, which gets even lower as the pulse width lengthens [17]. This might be overcome clinically by firing in contact mode. The increased ablation rate of Ho:YAG with higher frequencies (Table 1) is consistent with other reports [14, 18]. Whereas in clinical settings this could be explained by the decreased retropulsion, in this experimental setup where retropulsion does not play a role as aforementioned, this finding still needs to be elucidated. With TFL, nonetheless, higher frequencies did not have the same effect on the ablation rate. In fact, it dramatically drops when using 500 Hz, although power output was still 12–12.5 W. Furthermore, we tested a higher total output power [25 W (500 Hz/0.05 J; 1000 Hz/0.025 J)] (Table 1) and ablation rates significantly dropped, indicating that a very low pulse energy, despite a sizeable increase in pulse frequency, decreases the ablation effectiveness. Thus, very high frequencies, an acclaimed feature attributed to TFL, might be disputed, backed up further by the fact that such pulse frequencies are rarely reached in clinical practice [21]. Moreover, higher temperatures have been found for TFL compared to Ho:YAG, especially when using higher frequencies, although the temperature was below the thermal injury threshold. Recording the laser’s heat generation was beyond the scope of this study, but in clinical settings it is important to keep this in mind, and strategies such as access sheath or proper irrigation might help preventing thermal injury [22, 23].

These data support the TFL’s better performance in contact mode [11, 17]. Theoretically, if a higher and a lower pulse energy are compared, in the later, more energy will be absorbed by the water (between fiber tip and target). Using very low pulse energy, the rate of pulse energy effectively transmitted to the target (when not in contact mode) is likely very low as well. The use of very high frequency may partly balance the rate of lost energy per pulse. Similarly, it can be hypothesized that many pulses will be delivered on the same spot as a result of the high frequency. Hence, the distance will increase progressively after each pulse, as the first pulses will ablate the outermost surface, and a greater distance from the fiber tip is created, so that the energy for the following pulses will need to travel further. Further investigations exploring these interactions and the proposed hypotheses, as well as the role of the distance and repetition rate using extreme frequencies are mandatory.

A clinical study [8] found that VB modality was associated with faster lithotripsy (ureter: 20.4 vs. 16.1 min, *p* < 0.05; kidney: 28.7 vs. 19.8 min, *p* < 0.05) with no differences in total energy emitted (9.9 vs 10.7 kJ, *p* > 0.05; 13.5 vs 16.1 kJ, *p* > 0.05). Similarly, Rodriguez-Socarras et al. communicated higher ablation rate using VB technology in seven patients [9]; and Vizziello et al. [10], reported better performance and significantly lower lasering time using VB compared to standard pulse mode on BegoStones in a bladder training model. Although subjectively graded upon the user’s impression in the three aforementioned studies [8–10], less retropulsion was communicated in all.

Interestingly, the current work is the first wet-lab study providing robust evidence that the VB emission mode consistently increased the Ho:YAG ablation rate (Table 1, Fig. 3A), nearing those of TFL with 40 Hz/0.3 J settings. Table 2 shows an increase in the ablation rate when the VB mode is selected, even though the difference is statistically significant only for pulse energy values of 0.3 J and 0.5 J. For the other two Ho:YAG settings (with 0.8 J and 1 J pulse energy), the ablation rates tend to increase with respect to standard pulse, although statistical significance was not achieved. We hypothesized that VB mode could bring a speed advantage in the high frequency dusting technique, where a limited amount of pulse energy is used.

Noteworthy, retropulsion was not a factor in our experimental setup, therefore, the more efficient energy delivery itself may have improved the ablation rate, suggesting that VB improves the ablation rates not only by reducing retropulsion.

A similar emission mode called Moses™ technology (Lumenis, Yokneam, Israel) is commercially available, in which the energy is likewise released divided into two peaks, the first separating the fluid (Moses effect) so that the second is delivered to the target stone more effectively. This technology proved a higher ablation rate using different settings in vitro and less retropulsion both in vitro and in a porcine model [6]. Researchers tested the ablation volume (mm^3^) utilizing two fibers (200 and 365 µm) for 10 Hz/0.8 J (200 µm: 3.75 ± 0.30 vs 4.47 ± 0.30; 365 µm: 1.68 ± 0.30 vs 2.26 ± 0.19, both *p* = 0.01), 80 Hz/0.4 J (200 µm: 3.29 ± 0.45 vs 4.07 ± 0.23; 365 µm: 1.79 ± 0.18 vs 4.65 ± 0.52, *p* < 0.05), and 50 Hz/0.5 J (200 µm: 6.39 ± 1.21 vs 9.59 ± 0.66; 365 µm: 2.99 ± 0.12 vs 6.66 ± 0.39, both *p* < 0.01). These outcomes are consistent with the current report using VB, where higher frequencies led to greater improvement in the ablation rate (40 Hz/0.3 J: 114.35 [88.30–126.40] mg/min. Table 1, Fig. 3A). In addition, a randomized trial [7] allocated patients with upper tract stones to retrograde intrarenal surgery with either Moses™ or standard emission mode laser lithotripsy. The Moses™ technology arm resulted in significantly lower lasering time and retropulsion, with a comparable stone-free rate. There are no randomized trials for VB emission mode, in fact, there is a paucity of evidence, but from the few clinical [8, 9] and experimental [10] reports available to date, added to this report, it may be inferred that VB mode improves the ablation rate as compared to standard mode and even reaching paralleled effectiveness to TFL in certain settings. Nevertheless, further efforts comparing these modalities in the clinical settings are needed.

Some limitations are noted in this study that need to be considered before the landing of this data into the healthcare settings. First, the nature of this in vitro experimental settings portray a controlled environment that is certainly different from that faced in the clinical scenario. Additionally, we use only 272 µm core fiber and of a single laser fiber-stone distance (0.5 mm). Further studies using other fiber sizes and distances may provide further insights regarding the overall impact of pulse modulation and important information about how to correctly use both Ho:YAG and TFL lasers. Lastly, this study aimed to assess the ablation rates, but other important factors normally weighing in, such as retropulsion, fiber burn-back, irregular and heterogenous stones especially regarding their density, and the constantly changing stone-fiber distance, and the heat generation and thermal damage were not herein replicated.

## Conclusion

The ablation rate of TFL is higher than that of Ho:YAG, probably due to the wavelength itself. Moreover, the Virtual Basket™ emission mode, increased Ho:YAG ablation rates even reaching similar ablation rates of TFL in some modalities, and alike to other emission modes available such as the Moses™ technology. The elongation of the pulse width resulted in more effective ablation. Overall, very high frequencies with very low pulse energy dropped the ablation rate in TFL.

### Supplementary Information

Below is the link to the electronic supplementary material.

Supplementary file1 (TIFF 676 KB)

## Data Availability

The datasets analysed during the current study are available from the corresponding author on reasonable request.
