# Supplementary figures and images for: Understanding the ablation rate of Holmium:YAG and thulium fiber lasers. Perspectives from an in vitro study
Source: Urolithiasis. 2023 Jan 17;51(1):32. doi: 10.1007/s00240-022-01402-6 (PMC9845154; doi:10.1007/s00240-022-01402-6)

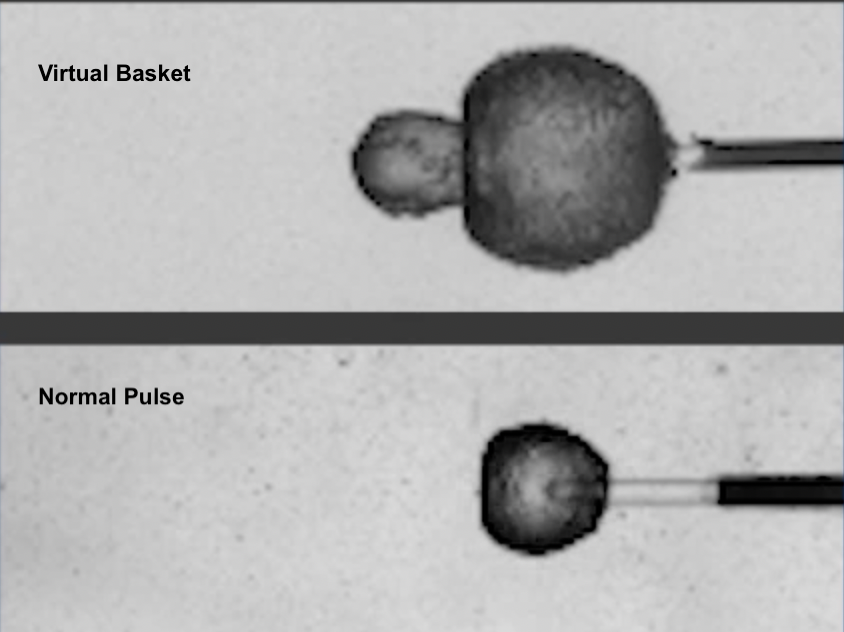

Supplement: Supplementary file 1 — Supplementary file1 (TIFF 676 KB) [file 240_2022_1402_MOESM1_ESM.tiff]
